# Agreement between Clinical History Method, Orbscan IIz, and Pentacam in Estimating Corneal Power after Myopic Excimer Laser Surgery

**DOI:** 10.1371/journal.pone.0123729

**Published:** 2015-04-08

**Authors:** Kaevalin Lekhanont, Manachai Nonpassopon, Khemruetai Wannarosapark, Varintorn Chuckpaiwong

**Affiliations:** Department of Ophthalmology, Ramathibodi Hospital, Mahidol University, Bangkok, Thailand; Bascom Palmer Eye Institute, University of Miami School of Medicine;, UNITED STATES

## Abstract

The purpose of this study was to investigate the agreement between the clinical history method (CHM), Orbscan IIz, and Pentacam in estimating corneal power after myopic excimer laser surgery. Fifty five patients who had myopic LASIK/PRK were recruited into this study. One eye of each patient was randomly selected by a computer-generated process. At 6 months after surgery, postoperative corneal power was calculated from the CHM, Orbscan IIz total optical power at the 3.0 and 4.0 mm zones, and Pentacam equivalent keratometric readings (EKRs) at 3.0, 4.0, and 4.5 mm. Statistical analyses included multilevel models, Pearson’s correlation test, and Bland-Altman plots. The Orbscan IIz 3.0-mm and 4.0 mm total optical power, and Pentacam 3.0-mm, 4.0-mm, and 4.5-mm EKR values had strong linear positive correlations with the CHM values (*r* = 0.90–0.94, P = <0.001, for all comparisons, Pearson’s correlation). However, only Pentacam 3.0-mm EKR was not statistically different from CHM (P = 0.17, multilevel models). The mean 3.0- and 4.0-mm total optical powers of the Orbscan IIz were significantly flatter than the values derived from CHM, while the average EKRs of the Pentacam at 4.0 and 4.5 mm were significantly steeper. The mean Orbscan IIz 3.0-mm total optical power was the lowest keratometric reading compared to the other 5 values. Large 95% LoA was observed between each of these values, particularly EKRs, and those obtained with the CHM. The width of the 95% LoA was narrowest for Orbscan IIz 3.0-mm total optical power. In conclusion, the keratometric values extracted from these 3 methods were disparate, either because of a statistically significant difference in the mean values or moderate agreement between them. Therefore, they are not considered equivalent and cannot be used interchangeably.

## Introduction

Cataract surgery in eyes with prior keratorefractive surgery can be challenging for ophthalmologists because of the difficulty in precisely calculating intraocular lens (IOL) power. Alterations in corneal shape after keratorefractive surgery can cause the inability of standard keratometry or computerized videokeratography to accurately measure anterior corneal curvature, the use of an invalid conventional keratometric index of refraction to calculate corneal power, and the inefficiency of most modern IOL formulas in predicting the effective lens position [1]. Several methods have been proposed to improve refractive outcomes either by estimating the true corneal power or making modifications to IOL formulas. The net corneal power can be obtained with techniques that rely on historical data or those that do not. Some surgeons consider the classic clinical history method (CHM), which requires preoperative information, to be the gold standard in calculating the corneal refractive power after keratorefractive surgery [2–6]. Nonetheless, the CHM method is not always applicable because pre-refractive surgery data are often missing or incomplete, and even if available, they can be simply imprecise and do not account for corneal changes after the laser vision correction procedure [7]. Additionally, no-history methods have been reported to give better results than methods using pre-refractive surgery keratometric values [8]. Therefore, a technology that enables the cataract surgeon to measure the correct corneal power directly, without the need for other assumptions should be an ideal way [9]. Theoretically, the anterior and posterior corneal curvatures and the corneal thickness have to be evaluated; the Gaussian optics formula can then be used to calculate the corneal refractive power [10–11]. Tomographers, including slit-scanning tomography (Orbscan IIz, Bausch & Lomb, New York, US), rotating Scheimpflug camera (Pentacam, Oculus, Wetzlar, Germany), and dual Scheimpflug (Galilei, Ziemer Ophthalmics AG, Port, Switzerland) systems have been used for this purpose [12–17]. Previous studies suggested that the change in corneal power after myopic laser in situ keratomileusis (LASIK) was best assessed with the total optical power map at the 4.0 mm zone in the Orbscan II system [12–14]. The equivalent keratometric readings (EKR) of the Pentacam system also provided a relatively good estimation of the central corneal power following keratorefractive surgery. The Pentacam 1.0, 2.0, 3.0, and 4.5-mm EKR have been found to closely correlate with the keratometric values derived from CHM [9,18–19]. Although other studies have compared the Orbscan measurements with the CHM and the Pentacam with the CHM separately, no studies to date have evaluated the agreement of the Orbscan IIz total optical power with the Pentacam EKR [1,9,14,18]. The aim of this study was to investigate the comparability of corneal power values obtained from the CHM, Orbscan IIz and Pentacam in post myopic keratorefractive eyes.

## Materials and Methods

Fifty five eyes of 55 patients who underwent either myopic LASIK or photorefractive keratectomy (PRK) at Ramathibodi Hospital, Bangkok, Thailand, from January 2010 to December 2012 were recruited into this prospective study. The study was approved by the ethics committee of Mahidol University School of Medicine and adhered to the tenets of the Declaration of Helsinki. All subjects gave written informed consent to participate in research. For participants younger than age 20, written parental consent was obtained. LASIK/PRK was performed using Technolas 217z Excimer Laser (Bausch & Lomb, New York, US) with optical zone of 6.0 mm. The Hansatome microkeratome (Bausch & Lomb, New York, US) was used to create a superior hinged flap. The cutting head (160μm) and the suction rings (8.5 mm or 9.0 mm) were selected depending on the white-to-white corneal diameter. The predicted residual stromal bed thickness was equal or greater than 250 μm. None had customized ablation. Inclusion criteria were age 18 years or older, postoperative corrected distance visual acuity of 20/25 or better, no flap or any sight-threatening complications, no other coexisting eye diseases, no other ocular surgeries, and good compliance with the study regimen and availability for the duration of the entire study period. Exclusion criteria were pregnant or lactating women, postoperative contact lens wear, and invalid or unreliable measurements. The minimum follow-up of 6 months was required to make certain that the postoperative refraction was stable to calculate corneal power according to the CHM. One eye of each patient was randomly selected by a computer-generated process. Uncorrected Snellen distance visual acuity, manifest and cycloplegic refraction, and postoperative Orbscan IIz and Pentacam analysis were measured at 6 months after surgery. These measurements were conducted by a single experienced technician for each instrument to minimize variation in the results.

Postoperative corneal power was estimated with the 3 following methods: the CHM, used as the benchmark for comparison; Orbscan IIz total optical power at the 3.0 and 4.0 mm zones; and Pentacam equivalent keratometric readings (EKRs) at 3.0, 4.0, and 4.5 mm. All participants had not developed cataract or received any lens extraction yet. Therefore, back-calculation of corneal power could not be done.

### Clinical history method

Preoperative and postoperative spherical equivalents were used to determine the change in manifest refraction spherical equivalent (MRSE) at the corneal plane. This value was then subtracted from the preoperative average keratometry to derive the postoperative corneal power. Keratometric readings before refractive surgery were obtained by automated keratometry.

### Orbscan IIz

The total optical power maps at the 3.0 and 4.0 mm zones were evaluated for each eye. The “Tools” menu and then “Analyze area-Statistics” for 3.0 and 4.0 mm of the central cornea were used to obtain corneal power values at both diameters. Although the refractive change after myopic LASIK has been shown to be best estimated using the 4.0-mm total optical power in the Orbscan II system [12–14], some studies found that using the smaller zones in calculation provided estimates closer to the correct postoperative corneal power [1,20–21]. Hence, the 3.0-mm total optical power was also chosen to be compared with the keratometry obtained by the CHM and Pentacam. All measurements were performed with fixation point centration.

### Pentacam

The measurements of EKRs shown in the Holladay report at 3.0, 4.0, and 4.5 mm were used. Even though the optimal zone for measuring the central corneal power in eyes that previously received corneal refractive surgery was determined to be 4.5 mm in the earlier study, EKRs at 3.0 and 4.0 mm have also been observed to be closely correlated with CHM [9,19]. Thus, 3.0-, 4.0-, and 4.5-mm EKRs were compared with CHM and Orbscan IIz values.

Only good-quality images from both devices were selected for analysis. Data were entered using the statistical software package STATA version 11.1 (Stata Corp, College Station, Texas, USA). After the estimated mean of each method was obtained, multilevel models were performed to evaluate the difference in estimated means between methods, along with 95% confidence intervals (CI). Pearson correlation coefficients between keratometric values derived from the various techniques were assessed. The between-instrument agreement in estimating the mean postoperative corneal power was analyzed by Bland-Altman method. The 95% limits of agreement (LoA) were defined as means ± 1.96 SD of the differences between the 3 types of calculation. The width of the 95% LoA in this study represented the range within which 95% of the differences between two measurements were likely to fall. *P*-values less than 0.05 were considered to be statistically significant.

## Results

The mean age of the patients was 33.11 ± 7.75 years (range, 16–54 years) and 79.41% were female. LASIK procedure was performed in most cases (51 eyes, 92.73%). The remaining 4 eyes (7.27%) underwent PRK. The mean MRSE before keratorefractive surgery was -5.2 ± 2.52 (range, -1.125 to -10.5) diopters (D). The mean postoperative MRSE was -0.3 ± 0.35 (range, -1.375 to 0.25) D. The average preoperative keratometric reading was 44.51 ± 1.46 (range, 42.05 to 47.55) D. The mean follow-up duration was 15.65 ± 8.99 (range, 6–36) months.

**Fig 1:**
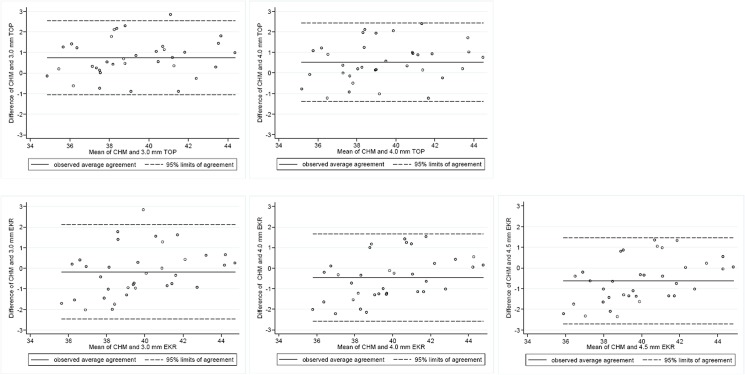
Bland-Altman plots showing differences in values of the estimated postoperative corneal power for comparisons between pairs of instruments. The 95% limits of agreement between the Orbscan IIz and CHM were wide. Agreement was even poorer when comparing the EKR at 3.0, 4.0, and 4.5 mm from Pentacam and CHM. CHM = clinical history method, TOP = total optical power from Orbscan IIz, EKR = equivalent keratometric readings from Pentacam.

Table 1 shows the postoperative corneal power measured from the 3 different methods, including their deviation from CHM. There were statistically significant differences among the estimated postoperative keratometric values derived from the 2 instruments and CHM (P = <0.001, multilevel models), except for the 3.0-mm EKR (P = 0.17). The corneal power values from both Orbscan IIz 3.0- and 4.0-mm total optical power maps tended to be flatter than those from CHM. Conversely, the average Pentacam EKRs at 4.0 and 4.5 mm diameters appeared to be steeper. The mean Orbscan IIz 3.0-mm total optical power was the lowest keratometric reading compared to the other 5 values. Strong linear positive correlations were found between each of these values obtained with Orbscan IIz or Pentacam, and historical-based values (*r* = 0.90–0.94, P = <0.001, for all comparisons, Pearson’s correlation). The Orbscan IIz 3.0-mm total optical power had the closest correlation with clinical history method (*r* = 0.94). The 95% LoA between the Orbscan IIz and CHM were wide (-1.05 to +2.53 D for 3.0-mm total optical power and -1.37 to +2.43 D for 4.0-mm total optical power). Agreement was even poorer when comparing the EKR at 3.0, 4.0, and 4.5 mm from Pentacam and CHM (-2.48 to +2.12 D, -2.57 to +1.65 D, and -2.72 to +1.47 D, respectively) (Fig 1).

**Table 1:** Differences between estimated postoperative corneal power (K) from Orbscan IIz, Pentacam, and clinical history method (CHM).

**Methods**	**K (Mean±SD)**	**ΔK** ^CHM^ **(Mean±SD)**	**95% CI of ΔK** ^CHM^ **(lower, upper)**	**P-value**	**95% LoA versus CHM**	**Pearson correlation (r)**
CHM^a^ (D)	39.62±2.65					
3.0 mm TOP (D)	38.88±2.50	0.74±0.91	0.49, 1.00	<0.001	-1.05 to 2.53	0.94
4.0 mm TOP (D)	39.09±2.44	0.53±0.92	0.27, 0.78	<0.001	-1.37 to 2.43	0.93
3.0 mm EKR (D)	39.80±2.26	-0.18±1.18	-0.78, 0.44	0.173	-2.48 to 2.12	0.90
4.0 mm EKR (D)	40.08±2.19	-0.46±1.08	-0.72, -0.20	<0.001	-2.57 to 1.65	0.92
4.5 mm EKR (D)	40.25±2.15	-0.62±1.07	-0.88, -0.37	<0.001	-2.72 to 1.47	0.92

^a^Estimated corneal power derived from CHM was used as a reference.

**Δ**K^CHM^ = K_CHM_-K_Orbscan IIz/Pentacam_, CI = confidence interval, LoA = limits of agreement, TOP = total optical power from Orbscan IIz, EKR = equivalent keratometric readings from Pentacam

**Fig 2:**
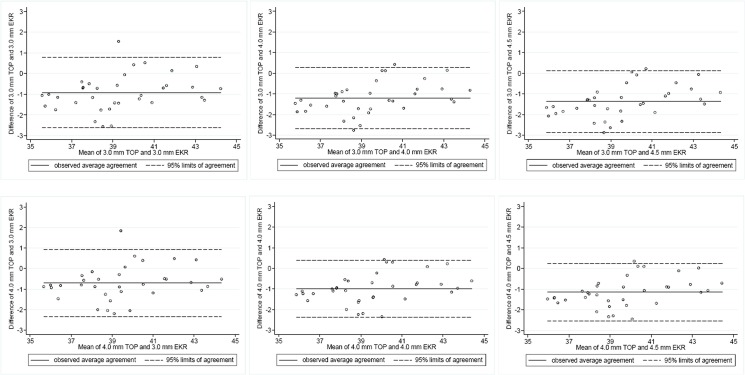
Bland-Altman plots showing differences in values of the estimated postoperative corneal power between Orbscan IIz total optical power (TOP) at 3.0 and 4.0 mm and Pentacam equivalent keratometric readings (EKR) at 3.0, 4.0, and 4.5 mm. Only a moderate level of agreement was found among these values.

The means of estimated postoperative keratometric readings from Orbscan IIz and Pentacam were also statistically different (Tables 2 and 3). Although the central 3.0- and 4.0-mm total optical powers correlated highly with EKR values, both of them were significantly lower than the EKRs and the Bland-Altman plot revealed only a moderate level of agreement among these values (Fig 2).

**Table 2:** Differences between estimated postoperative corneal power (K) from 3-mm Orbscan IIz and Pentacam.

**Methods**	**K (Mean±SD)**	**ΔK** ^Orb 3^ **(Mean±SD)**	**95% CI of ΔK** ^Orb 3^ **(lower, upper)**	**P-value**	**95% LoA versus 3-mm TOP Orbscan IIz**	**Pearson correlation(r)**
3.0 mm TOP^a^ (D)	38.88±2.50					
3.0 mm EKR (D)	39.80±2.26	-0.92±0.87	-1.11, -0.73	<0.001	-2.62 to 0.78	0.93
4.0 mm EKR (D)	40.08±2.19	-1.20±0.76	-1.39, -1.01	<0.001	-2.69 to 0.28	0.97
4.5 mm EKR (D)	40.25±2.15	-1.37±0.76	-1.56, -1.18	<0.001	-2.85 to 0.12	0.96

^a^Estimated corneal power derived from 3.0 mm TOP was used as a reference.

**Δ**K^Orb 3^ = K_Orbscan IIz at 3-mm—_K_Pentacam_, CI = confidence interval, LoA = limits of agreement, TOP = total optical power from Orbscan IIz, EKR = equivalent keratometric readings from Pentacam

**Table 3:** Differences between estimated postoperative corneal power (K) from 4-mm Orbscan IIz and Pentacam.

**Methods**	**K (Mean±SD)**	**ΔK** ^Orb 4^ **(Mean±SD)**	**95% CI of ΔK** ^Orb 4^ **(lower, upper)**	**P-value**	**95% LoA versus 4-mm TOP Orbscan IIz**	**Pearson correlation(r)**
4.0 mm TOP^a^ (D)	39.09±2.44					
3.0 mm EKR (D)	39.80±2.26	-0.70±0.83	-0.89, -0.52	<0.001	-2.33 to 0.92	0.94
4.0 mm EKR (D)	40.08±2.19	-0.99±0.71	-1.17, -0.80	<0.001	-2.38 to 0.40	0.96
4.5 mm EKR (D)	40.25±2.15	-1.15±0.70	-1.33, -0.97	<0.001	-2.53 to 0.23	0.96

^a^Estimated corneal power derived from 4.0 mm TOP was used as a reference.

**Δ**K^Orb 4^ = K_Orbscan IIz at 4-mm—_K_Pentacam_, CI = confidence interval, LoA = limits of agreement, TOP = total optical power from Orbscan IIz, EKR = equivalent keratometric readings from Pentacam

## Discussion

In this study, we assessed the corneal power measurements provided by the Orbscan IIz and Pentacam to evaluate whether these values were comparable and could be reliably employed for IOL power calculation after keratorefractive surgery when complete perioperative data were not available. Traditionally, the CHM has been considered to be the gold standard to estimate the actual corneal power postrefractive surgery and thus adopted as the benchmark for comparisons in several studies, including our study [2–6,18,22]. However, increasing evidence suggested that the accuracy of CHM might not be the best because the relevant data could be imprecise or unstable either due to inaccurate measurements or interval changes in the corneal curvature or lens power and clarity [23–24]. Moreover, the repeatability of the CHM has not been studied [18]. Therefore, the most practical and safest methods of determining IOL power postrefractive surgery should be independent of historical data [23,25–26]. More advanced techniques that allow one to measure the curvature of both anterior and posterior corneal surfaces, such as optical coherence tomography and other corneal tomography systems including slit-scanning tomography, rotating slit Scheimpflug-camera, and dual- Scheimpflug, are supposed to be methods that best estimate the true corneal power and can be used as an alternative to the CHM. There have been several promising reports of measuring keratometric values using the Orbscan IIz and Pentacam systems to calculate IOL power [13,20–21,27]. Although both instruments can directly evaluate the posterior corneal curvature, they use different operating principles. The Pentacam has superior depth of focus and use rotational scanning, which may fundamentally outperform the Orbscan IIz device [23]. Nonetheless, there are limited data comparing the keratometric values from Pentacam and Orbscan IIz.

Our results demonstrated that all Orbscan IIz 3.0-mm and 4.0 mm total optical power, and Pentacam 3.0-mm, 4.0-mm, and 4.5-mm EKR values had strong statistical correlation with the CHM values. However, only Pentacam 3.0-mm EKR was not statistically different from CHM. The mean 3.0- and 4.0-mm total optical powers of the Orbscan IIz were significantly lower than the values derived with the CHM, while the average EKRs of the Pentacam at 4.0 and 4.5 mm were significantly higher. In addition, considerably large 95% LoA was observed between each of these values, particularly EKRs, and those obtained with the CHM. The width of the 95% LoA was narrowest for Orbscan IIz 3.0-mm total optical power. These findings are similar to those seen in previous study, in which the EKRs at smaller zones (1.0, 2.0, and 3.0 mm) were the only Pentacam measurements not statistically different from the corneal power values calculated from the CHM and the 95% LoA were wide [18]. A few studies have also revealed that the mean EKR in the 4.5 mm optical zone overestimated the true corneal power and this effect increased as the cornea became flatter [28]. On the other hand, some prior studies found that the EKR at 4.5 mm most closely correlated with CHM, provided narrower 95% CI and 95% LoA than other Pentacam values, and gave a relatively good estimation of the central corneal power after keratorefractive surgery at least for a 6.0–7.0-mm optical zone [9,19]. Possible explanations for the discrepancies between the results of these studies are differences in intended correction, laser platform, surgical procedure, size of optical zone, and ablation profile. Our study included patients with low to high myopia and LASIK/PRK was done to correct an average MRSE of -5.2 D which resembled that of previous report preferred smaller EKR (-5.1 D) [18]. Meanwhile, the study that suggested larger EKR recruited only low to moderate myopic patients and thus the intended correction was low (-3.6 D) [9]. Consequently, the appropriate EKR for each individual may vary, depending on the type and amount of ablations. Although the EKR values represent the central corneal power more precisely than the keratometric readings obtained with standard topographers and keratometers, they should only be used to calculate the IOL power in formulas that are optimized for EKR, like the BESSt or Holladay-2 formulas [29]. Moreover, the Scheimpflug power measurements were fairly steeper than the other values. This could probably lead to underestimation of the IOL power and a consecutive postoperative hyperopia. Recently, other Pentacam values, such as true net power (TNP) and total corneal refractive power (TCRP), showed better performance in assessing postoperative corneal power after corneal refractive surgery in patients without a clinical history [21–22,27,30–31]. The TCRP also seemed to accurately reflect the surgically induced refractive change after myopic excimer laser surgery [31]. Therefore, TNP and TCRP, as well as additional refinement of the EKR software deserve further study.

The Orbscan system combines Placido disk and slit-scanning technology to directly measure both elevation and curvature of the anterior and posterior corneal surfaces [14]. Despite taking advantages of both technologies, the Orbscan has its own drawbacks. Corneal opacity or irregularity may obscure imaging of the posterior cornea and introduce artifacts into total corneal power calculations. The reliability of posterior corneal measurements with the Orbscan has also not been fully established, especially for post-refractive surgery eyes in which image-detection programs may have difficulty in identifying the posterior aspect of the corneal slit beam because of the change in contrast [14]. Nevertheless, the posterior surface refracts light from the cornea (n = 1.376) to the aqueous humor (n = 1.336), yielding less refractive power than the anterior corneal surface. Accordingly, potential errors of the posterior surface might be reduced in the conversion to diopters [14]. The total optical power map in Orbscan applies ray tracing method and analyzes the cornea’s total effective power for the area of interest using Snell’s law. This is believed to be precise in the setting of excimer laser ablation [14]. We found that the 3.0-mm total optical power had the highest agreement with the corneal power determined by CHM and it was significantly flatter on average than the other parameters. Hence, using this value to calculate the IOL power for cataract surgery might result in less hyperopic surprise following operation.

There are many reasons of the differences in corneal power measurements among the 3 methods. The CHM uses only preoperative and postoperative variables derived from manifest refraction and automated keratometry to calculate the total corneal power, regardless of corneal changes after the laser vision correction procedure, while both Pentacam and Orbscan estimate the corneal power by directly measuring the anterior and posterior corneal curvatures, and the corneal thickness postoperatively. Nonetheless, the difference in performance between rotating Scheimpflug photography and scanning-slit topography may account for the different estimated corneal powers between these two instruments. In the assessment of image quality, the Pentacam and Orbscan images showed similar signal difference-to-noise ratios of cornea versus background, however, the intensity profiles showed steeper corneal edge depiction by the Pentacam, thereby potentially contributing to a decrease in detection errors as a result of less blurred images [32]. Additionally, Pentacam and Orbscan IIz had a measurable difference in posterior elevations above the best-fit sphere, despite similar radii of curvature. Orbscan appeared to determine the posterior elevation above the best-fit sphere higher than the Pentacam [33]. This difference is probably due to the difference in operating principles between the 2 systems, as the scanning-slit topographer estimates the central 3 mm, whereas the Scheimpflug images the cornea directly [33]. Other mechanisms that could explain the disparity include density of the pixels, the software used to image the cornea, and clarity of the cornea or tear film [33]. Also, although Scheimpflug photography and scanning-slit topography had good within-rater repeatability and between-rater reproducibility for measuring corneal shape, Scheimpflug photography demonstrated slightly better repeatability and reproducibility characteristics and might be preferred in a clinical setting [34]. However, with the lack of a true gold standard, the interpretation of differences between the 3 methods is significant and we cannot provide an answer to a question of which method would be more valid. Too much emphasis should not be placed on the mean difference between these methods especially when there are both positive and negative differences. In this case, the mean difference may be misleadingly low [35]. On the other hand, the 95% LoA provide an estimate for the interval in which 95% of the actual differences between measurements will lie [35]. For instance, although there was the good concordance between postoperative corneal power calculations obtained with CHM, Orbscan IIz, and Pentacam in our study, the wide LoA arguably exceed the clinically acceptable range of agreement. The importance of the study is to understand that when using the machines a direct comparison may not be possible. Because of some level of inevitable uncertainty, particularly in non-virgin eyes, surgeons should become adept at interpreting the machines they use routinely and apply several well established methods combined with clinical judgment to improve consistency in refractive outcomes [23].

There are some limitations of this study. First, the sample size was relatively small. Because of this, although other factors such as age and diopter could have an influence on keratometric reading, we neither divided the patients into different age groups nor separately examined eyes with lower and higher amount of myopic correction. Second, all eyes were operated on with the same laser platform, different outcomes could be found with other lasers and ablation profiles. Third, the follow-up time was short. Patients with a long follow-up, complex cornea, topography- or wavefront- guided ablation, decentered ablation, or very small optical zones were not included in the study. Our findings might be valid only for uncomplicated cases with a short follow-up (6–36 months). Also, there was just a single experienced technician for each instrument; it might increase the operator error. Fourth, our comparison was limited to 3 main parameters which were CHM, total optical power, and EKR. This is because our primary intention was to compare keratometric values from 2 different machines in the same patient and find out which matches the corneal power value obtained with CHM and is most proper to be used with the available current theoretical IOL formulas (SRK/T, Holladay, Hoffer Q). Finally, back-calculation of corneal power after cataract surgery and IOL implantation was not done. Having this value as the benchmark of true corneal power should increase the accuracy of the results. Furthermore, besides keratometric reading, other data should have been collected to measure the agreement among the instruments in order to increase the credibility of this study. Future larger studies are necessary to compare several more methods of estimating corneal power after excimer laser surgery, and evaluate hyperopic LASIK/PRK group and patients who have had surgery for a long time.

## Conclusions

In conclusion, the keratometric values extracted from Orbscan IIz 3.0- and 4.0-mm total optical power maps, Pentacam 3.0-, 4.0-, and 4.5-mm EKR maps, and CHM were disparate, either because of a statistically significant difference in the mean values or moderate agreement between them. Therefore, these 3 methods are not considered equivalent and cannot be used interchangeably. Since the lack of a gold standard for corneal power measurement following keratorefractive surgery and thus unknown true corneal power, it is not possible to conclude which device or parameters provides the most accurate value. Both Orbscan and Pentacam have distinct advantages of simplicity and independence of historical data, however, because of wide LoA, they should be used cautiously as a sole instrument for determining the corneal power.
